# Alterations of Glycan Composition in Aerobic Granular Sludge during the Adaptation to Seawater Conditions

**DOI:** 10.1021/acsestwater.3c00625

**Published:** 2023-12-27

**Authors:** Le Min Chen, Sunanda Keisham, Hiroaki Tateno, Jitske van Ede, Mario Pronk, Mark C. M. van Loosdrecht, Yuemei Lin

**Affiliations:** † Department of Biotechnology, Delft University of Technology, Van der Maasweg 9, 2629 HZ Delft, The Netherlands; ‡ Cellular and Molecular Biotechnology Research Institute, National Institute of Advanced Industrial Science and Technology (AIST), Central 6, 1-1-1 Higashi, Tsukuba, Ibaraki 305-8566, Japan; § Royal HaskoningDHV, Laan 1914 35, Amersfoort 3800 AL, The Netherlands

**Keywords:** glycans, glycoproteins, aerobic granular sludge, extracellular polymeric substances, lectin microarray

## Abstract

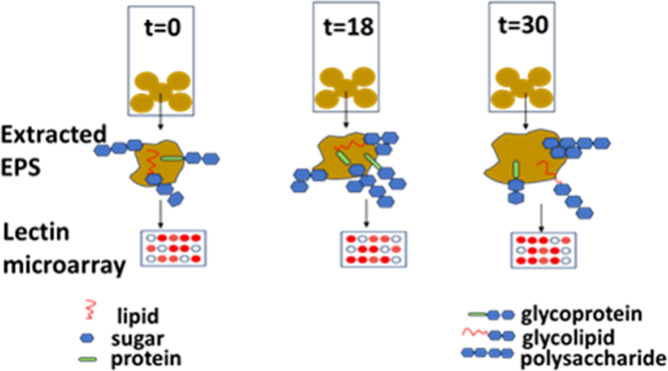

Bacteria can synthesize a diverse array of glycans, being found attached to proteins and lipids or as loosely associated polysaccharides to the cells. The major challenge in glycan analysis in environmental samples lies in developing high-throughput and comprehensive characterization methodologies to elucidate the structure and monitor the change of the glycan profile, especially in protein glycosylation. To this end, in the current research, the dynamic change of the glycan profile of a few extracellular polymeric substance (EPS) samples was investigated by high-throughput lectin microarray and mass spectrometry, as well as sialylation and sulfation analysis. Those EPS were extracted from aerobic granular sludge collected at different stages during its adaptation to the seawater condition. It was found that there were glycoproteins in all of the EPS samples. In response to the exposure to seawater, the amount of glycoproteins and their glycan diversity displayed an increase during adaptation, followed by a decrease once the granules reached a stable state of adaptation. Information generated sheds light on the approaches to identify and monitor the diversity and dynamic alteration of the glycan profile of the EPS in response to environmental stimuli.

## Abstract 2

This study provides insight into the approaches to identify and monitor the diversity and dynamic alteration of the glycan profile of the extracellular polymeric substances in response to environmental stimuli.

## Introduction

Carbohydrates constitute the most structurally diverse class of natural products and can serve many functions in cells and organisms.^1^ Glycans refer to carbohydrate chains that can be free or attached to proteins or lipids to form simple or complex glycoconjugates.^2^ Glycans participate in almost every biological process.^3^ In addition to forming important structural features, the glycans of glycoconjugates modulate or mediate a wide variety of functions, such as cell adhesion, recognition, receptor activation, or signal transduction in animal and plant cells.^4^

Bacteria can synthesize a diverse array of glycans, being found attached to proteins and lipids, or as loosely associated polysaccharides to the cells.^1^ The precise role of these glycans in bacterial symbiosis and cell–cell and cell–environment interactions is just beginning to be understood. Most of bacterial glycans are located at the surface of cells, deposited in the extracellular space and attached to soluble signaling molecules.^1^ In this respect, when biofilm is formed, as the extracellular polymeric substances (EPS) are the components that form the matrix wherein the microorganisms are embedded, bacterial glycans are one of the important components of the EPS. However, EPS are frequently reported consisting of proteins (structural proteins and enzymes), polysaccharides, nucleic acids, and lipids,^5^ which overlooks the possibility that proteins and polysaccharides and lipids and polysaccharides in EPS may present not only as separate components but also in various forms of glycoconjugates.^6^ Moreover, the frequently used EPS characterization methods (e.g., colorimetric methods) only allow for characterization of the separate classes of molecules but provide little insight into the glycoconjugates.

At present, one of the proven effective methods for EPS glycoconjugate analysis is fluorescence lectin bar-coding (FLBC).^7^ These lectins can bind to specific carbohydrate regions, allowing for the screening of glycoconjugates in a hydrated biofilm matrix. This method has been successfully applied to the analysis of a few different types of biomass, such as saline aerobic granular sludge, anaerobic granular sludge, anammox granular sludge, and “*Candidatus* Accumulibacter phosphatis” enrichment.^8−10^ Glycans, with sugar residues including sialic acids, mannose, galactose, *N*-acetyl-galactosamine, and *N*-acetyl-glucosamine, were found in the EPS of those biomasses.^11,12^ It is worth pointing out that information provided by this method only reflects the composition of the carbohydrates; it is still unclear whether these carbohydrates are attached to proteins, lipids, or simply as polysaccharides. Hence, to unravel the complete glycan profile of the EPS in biofilms, it is significantly important to establish methodologies to identify glycoconjugates such as glycoproteins and glycolipids.

Protein glycosylation is the covalent attachment of single sugars or glycans to select residues of proteins. It is one of the common yet most complex post-translational modifications. Protein glycosylation has profound effects on protein function and stability.^13^ Historically, glycosylation of proteins was used to be considered to occur exclusively in eukaryotes; only recently it is accepted that prokaryotes can also perform (complex) protein glycosylation.^14^ The glycosylation of prokaryotic proteins is far less studied, and most of the research focuses on specific pathogenic bacteria. Regarding a few studies on the glycoproteins in environmental samples, such as the glycoproteins in the EPS of anammox granular sludge, mass spectrometry was performed.^15^ While this approach enables deciphering the structure of glycans derived from glycoproteins, it is not amenable to adaptation to a high-throughput platform.^16^ This brings a severe bottleneck in monitoring the diversity and dynamic alteration of the glycan profile. Especially, given that such diverse structures are important interfaces between bacteria and the environment. Thus, the major challenge in glycan research in the environmental field lies in developing high-throughput and comprehensive characterization methodologies to elucidate the structure and monitor the change of glycosylation.

To this end, in the current research, the dynamic change of the glycan profile of a few EPS samples was monitored by Gas chromatography–mass spectrometry (GC-MS) and high-throughput lectin microarray as well as the sialylation and sulfation analysis. Those EPS samples were extracted from aerobic granular sludge collected at different stages during its adaptation to seawater conditions. The information generated sheds light on the approaches to identify and monitor the diversity and dynamic alteration of the glycan profile of the biomass in response to environmental stimuli.

## Experimental Methods

### Reactor Operation

Seawater-adapted aerobic granular sludge was cultivated in a 2.8 L bubble column (6.5 cm diameter) as a sequencing batch reactor (SBR) adapted from de Graaf et al.^12^ The reactor was inoculated with aerobic granular sludge cultivated in a lab-scale reactor with glycerol as the carbon source under freshwater condition.^17^ The temperature was controlled at 20 °C, and the pH was controlled at pH 7.3 ± 0.1 by dosing 1.0 M NaOH or 1.0 M HCl. The DO was controlled at 2 mg of O_2_/mL (80% saturation). Reactor cycles consisted of 60 min of anaerobic feeding, 170 min of aeration, 5 min of settling, and 5 min of effluent withdrawal. Artificial seawater was gradually introduced for 13 days until a concentration of 35 g/L was reached.

To investigate the glycan profile of the extracellular polymeric substances of aerobic granules during their adaptation to seawater, granules were collected at three different time slots: t0, t18, and t30. The sample at t0 refers to the inoculum. The sample at t18 was collected 18 days after the reactor started (5 days after the seawater concentration in the reactor achieved 35 g/L; the SRT in the reactor was not controlled). The sample at t30 was taken 30 days after the reactor start (the SRT in the reactor was controlled as 13.6 days), representing a relatively stable state of seawater-adapted granules.

The organic and ash fractions of the biomass were determined according to the standard methods after washing the granules three times with demi-water.^18^ For EPS extraction and characterization, the granules were lyophilized immediately and stored at room temperature.

### Microbial Community Analysis by Fluorescent In Situ Hybridization (FISH)

To investigate the microbial community, fluorescent in situ hybridization (FISH) was performed. The handling, fixation, and staining of samples were performed as described in Bassin et al.^19^ A mixture of EUB338, 13 EUB338-II, and EUB338-III probes were used to stain all of the bacteria.^20^ A mixture of PAO462, PAO651, and PAO846 probes (PAOmix) was used for visualizing polyphosphate accumulating organisms (PAOs).^21^ A mixture of GAOQ431 and GAOQ989 probes (GAOmix) was used to target glycogen accumulating organisms (GAOs).^21^ The samples were examined with a Zeiss Axioplan 2 epifluorescence microscope equipped with filter sets 26 (bp 575e625/FT645/bp 660e710), 20 (bp 546/12/FT560/bp 575e640), and 17 (bp 485/20/FT 510/bp 5515e565) for Cy5, Cy3, and fluos, respectively.

### EPS Extraction from Aerobic Granular Sludge

Lyophilized granules were extracted in 0.1 M NaOH (1% VS w/v) for 30 min at 80 °C while being stirred at 400 rpm. The solution was cooled and centrifuged at 4000*g* for 20 min at 4 °C. The supernatant was collected and subsequently dialyzed against demi-water overnight in dialysis tubing with a molecular weight cutoff of 3.5 kDa MWCO (Snakeskin, ThermoFisher Scientific, Landsmeer). The dialyzed EPS solution was lyophilized and stored at room temperature until further analysis.

### EPS Characterization

#### Glycosyl Composition Analysis by TMS Method

Glycosyl composition analysis of the extracted EPS was performed at the Complex Carbohydrate Research Center (CCRC, University of Georgia) by combined GC/MS of the O-trimethylsilyl (TMS) derivatives of the monosaccharide methyl glycosides produced from the sample by acidic methanolysis. These procedures were carried out as previously described in Santander et al.^22^ In brief, lyophilized EPS aliquots of 300 μg were added to separate tubes with 20 μg of inositol as the internal standard. Methyl glycosides were then prepared from the dry sample following the mild acid treatment by methanolysis in 1 M HCl in methanol at 80 °C (16 h). The samples were re-N-acetylated with 10 drops of methanol, 5 drops of pyridine, and 5 drops of acetic anhydride and were kept at room temperature for 30 min (for detection of amino sugars). The sample was then per-o-trimethylsilylated by treatment with Tri-Sil (Pierce) at 80 °C (30 min). These procedures were carried out as described by Merkle & Poppe.^23^ GC/MS analysis of the per-o-trimethylsilyl methyl glycosides was performed on an AT 7890A gas chromatograph interfaced to a 5975B MSD mass spectrometer, using a Supelco EC-1 fused silica capillary column (30 m × 0.25 mm ID) and the temperature gradient shown in Table 1.

**Table 1:** Temperature Program for GC-MS Analysis for the TMS Method

	**rate** (°C/min)	**value (°C)**	**hold time (min)**	**run time (min)**
initial		80	2	2
ramp 1	20	140	2	7
ramp 2	2	200	0	37
ramp 3	30	250	5	43.7

#### Sulfated Glycosaminoglycan Assay

Detection and quantification of sulfated glycosaminoglycans (sulfated GAGs) in the extracted EPS were performed with the Blyscan sulfated glycosaminoglycan assay (Biocolor, Carrickfergus, UK), according to the manufacturer’s instructions. Samples (2–5 mg) were digested with 1 mL of papain protein digestion solution at 65 °C for 3 h at 300 rpm (Sigma-Aldrich, Zwijndrecht, Netherlands). The supernatant was recovered after centrifugation at 10,000*g* for 10 min. 50 μL of sample was then added to 1 mL of 1,9-dimethyl-methylene blue (DMMB) dye reagent. Sulfated GAGs positive components bind and precipitate with the dye and are subsequently isolated and resolubilized. The concentration of sulfated GAGs was measured with a multimode plate reader at 656 nm (TECAN Infinite M200 PRO, Switzerland) as chondroitin sulfate equivalents. Lastly, the distribution of N-linked and O-linked sulfates in the samples was measured by performing nitrous acid cleavage according to the manufacturer’s instructions prior to sulfated GAGs quantification.

#### Nonulosonic Acid Analysis with Mass Spectroscopy

Detection of nonulosonic acids (NulOs) in the extracted EPS was done according to the approach described by Kleikamp et al. (2020). In short, lyophilized EPS fractions were hydrolyzed by 2 M acetic acid for 2 h at 80 °C and dried with a Speed Vac concentrator. The released NulOs were labeled using DMB (1,2-diamino-4,5-methylenedioxybenzene dihydrochloride) for 2.5 h at 55 °C and analyzed by reverse phase chromatography Orbitrap mass spectrometry (QE plus Orbitrap, ThermoFisher Scientific, Bleiswijk, Netherlands).

#### Glycan Profiling of Glycoproteins by Lectin Microarray Analysis

High-density lectin microarray was generated according to the method described.^24^ 0.4 μg of EPS was labeled with Cy3-N-hydroxysuccinimide ester (GE Healthcare), and excess Cy3 was removed with Sephadex G-25 desalting columns (GE Healthcare). Cy3-labeled proteins were diluted with probing buffer [25 mM tris-HCl (pH 7.5), 140 mM NaCl, 2.7 mM KCl, 1 mM CaCl_2_, 1 mM MnCl_2_, and 1% Triton X-100] to 0.5 μg/mL and were incubated with the lectin microarray at 20 °C overnight. The lectin microarray was washed three times with probing buffer, and fluorescence images were captured using a Bio-Rex scan 200 evanescent-field-activated fluorescence scanner (Rexxam Co. Ltd., Kagawa, Japan).

The obtained signals were mean-normalized, and ANOVA test was performed using IBM SPSS Statistics 24.0 to identify lectins with significantly different intensities between the three samples. Heatmap of the lectins with significant intensities (*p* < 0.05) was performed using the Rpackage Pheatmap (version 1.0.12) on RStudio (version 4.2.2). Student′s *t* test was performed using IBM SPSS for statistical analysis between EPSt18 and EPSt30 to obtain the t-value.

## Results

### Reactor Operation and Microbial Community in Seawater-Adapted Aerobic Granular Sludge

**Figure 1:**
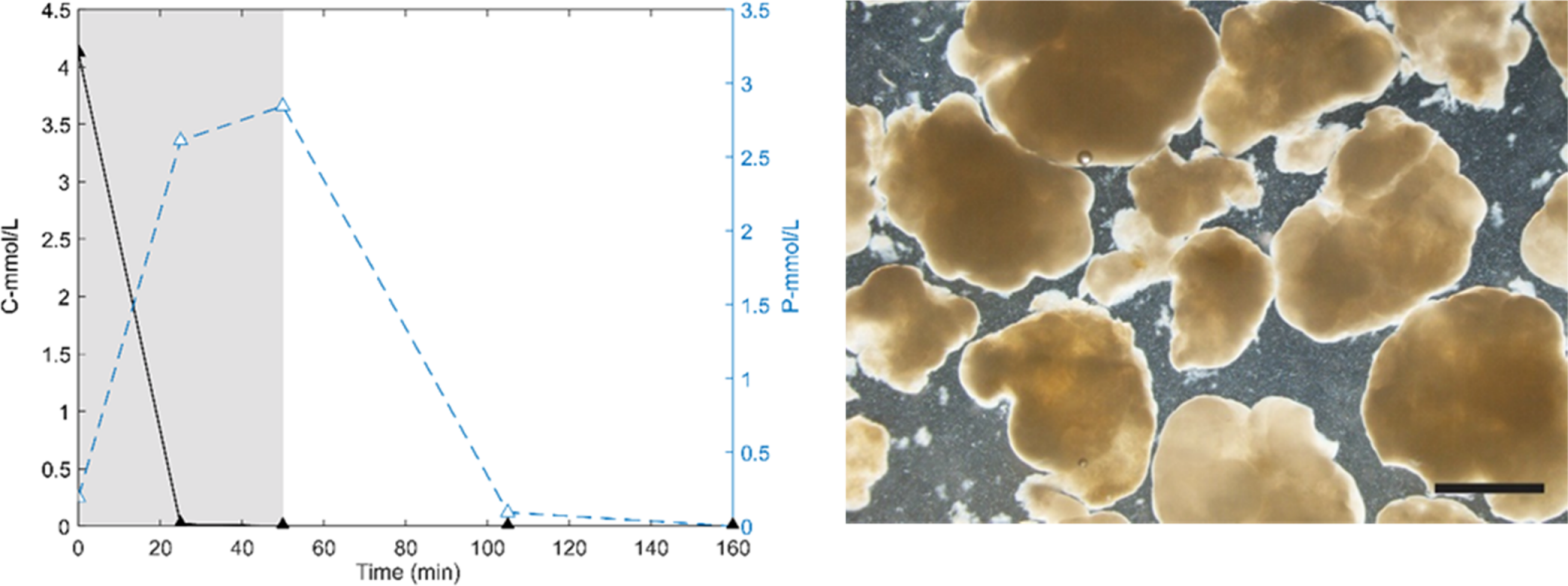
Left: reactor profile of a typical cycle of seawater-adapted aerobic granular sludge (t30). The uptake of the carbon source, acetate, is expressed in C-mmol/L indicated with black-filled triangles. The release and uptake of phosphate are indicated with blue open triangles. The anaerobic phase is indicated by the shaded area (50 min), followed by the aerobic phase (110 min). Right: the morphology of granules. No visual differences were observed among the three samples. The scale bar is 1 mm.

An aerobic granular sludge reactor was inoculated with granular sludge from the other lab reactor (with glycerol as a carbon source (t0)). Acetate was used as a carbon source to enrich specifically for phosphate accumulating organisms (PAOs).^12^ The salinity in the reactor was stepwise increased until 35 g/L of seawater was reached. After 7 days, complete acetate and phosphate removal were observed. Granular sludge samples were collected on the 18th and 30th days after the start of the reactor. The typical reactor profiles of t0, t18, and t30 show similar trends in acetate uptake and phosphate removal (Figure 1). During the anaerobic phase, acetate was taken up and a phosphate release was found to be up to 2.72 Pmmol/L. The reactor’s biomass concentration was roughly constant at around 7 g VSS/L with a VSS/TSS of around 76%. The morphology of the granules is shown in Figure 1. No visual differences were observed among the three samples.

**Figure 2:**
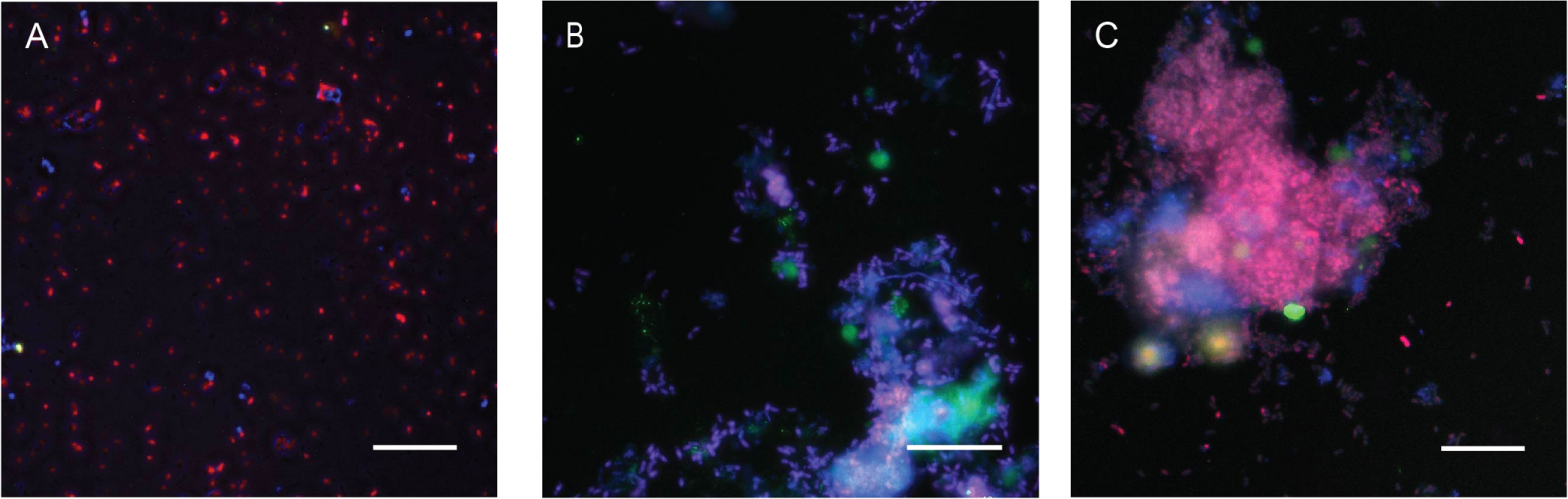
Fluorescence in situ hybridization (FISH) images of aerobic granular sludge (AGS) stained by EUB338 (Cy5/blue, eubacteria), PAO651 (A) or PAOmix (B and C, Cy3/red), and GAO (fluos/green). Magenta color is an overlap between eubacteria (blue) and PAOmix (red). Scale bar equals 20 μm. A: inoculum (t0); B: granules 18 days after inoculation (t18); and C: granules 30 days after inoculation (t30).

According to the FISH analysis, PAO was the dominant microorganism in the three granule samples (Figure 2). While the abundance of glycogen accumulating organisms (GAOs) was much lower than that of PAO. Comparatively, the abundance of GAO in granules collected at t18 (Figure 2A) seemed relatively higher than that in granules collected at t0 and t30 (Figure 2B,C). It was also observed that the size of the microcolony of PAO was much bigger in granules at t30 than in granules at t0 and t18.

### EPS Extraction and Characterization

The extracted EPS has the same yellow color as the aerobic granules. The yield of EPS at t0, t18, and t30 was 308 ± 117, 385 ± 82 mg/g, and 640 ± 42 (VSS ratio), with VS/TS ratios of 69, 70, and 86%, respectively. Apparently, during the adaptation to seawater conditions, more EPS, which can be extracted with NaOH, was produced.

#### Glycosyl Composition

The glycosyl composition of the extracted EPS is listed in Table 2, and the GC-MS chromatogram is included in the Supporting Information. The total carbohydrate amount increased from EPS_t0_ to EPS_t30_ (Table 1). Glucose, rhamnose, mannose, fucose, and galactose were found to be the main components of all samples. The relative molar ratio of each sugar monomer varied among samples, with glucose as the most abundant monomer. Xylose and *N-*acetyl glucosamine were also found in the seawater-cultured samples, while only the inoculum contained arabinose. Additionally, an unknown sugar was detected in all of the samples at about 29.3 min (marked by asterisk* in GC spectrum in the Supporting Information). Thus, based on sugar composition, there is a clear difference between the inoculum and seawater-grown granular sludge EPS.

**Table 2:**
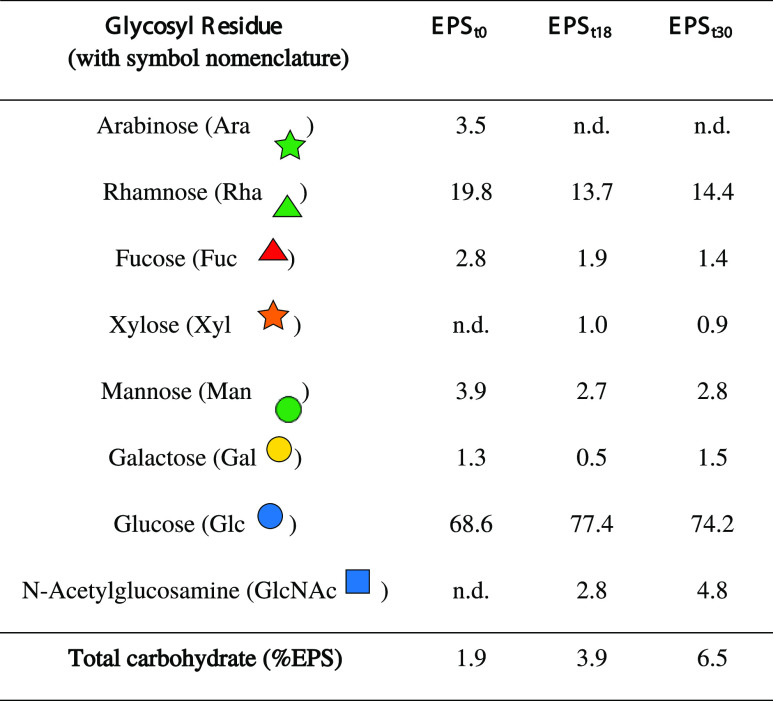
Glycosyl Composition of the Extracted EPS (Relative Mole and Total Carbohydrate Percentage. Monomer Symbol Nomenclature Is based on ref (25).)

#### NulOs and Sulfated Glycosaminoglycan-like Polymers

Glycoconjugate modifications with acidic groups such as sulfate (sulfation) and/or sialic acid (sialylation) on the glycans are common phenomena in the extracellular matrix of eukaryotes. Recently, these two glycoconjugate modifications (sulfation and sialylation) were found to be widely distributed in the EPS of granular sludge as well.^26^ In order to investigate the influence of seawater conditions on sulfation and sialylation, the same analysis was performed on the extracted EPS samples.

To identify which kinds of nonulosonic acids (NulOs, sialic acids is one type of nonulosonic acids) are present in the granules, mass spectrometry (MS) was applied. NulOs were detected in the form of *N-*acetyl neuraminic acid (NeuAc) and pseudaminic acid/legionaminic acid (Pse/Leg, which are also referred to as bacterial sialic acids in the literature. These two monomers have the same molecular weight and cannot be differentiated by MS). Hence, there are two different kinds of NulOs in all of the EPS samples. These NulOs could be part of glycoconjugates, including glycolipids, glycoproteins, and capsular polysaccharides.

**Figure 3:**
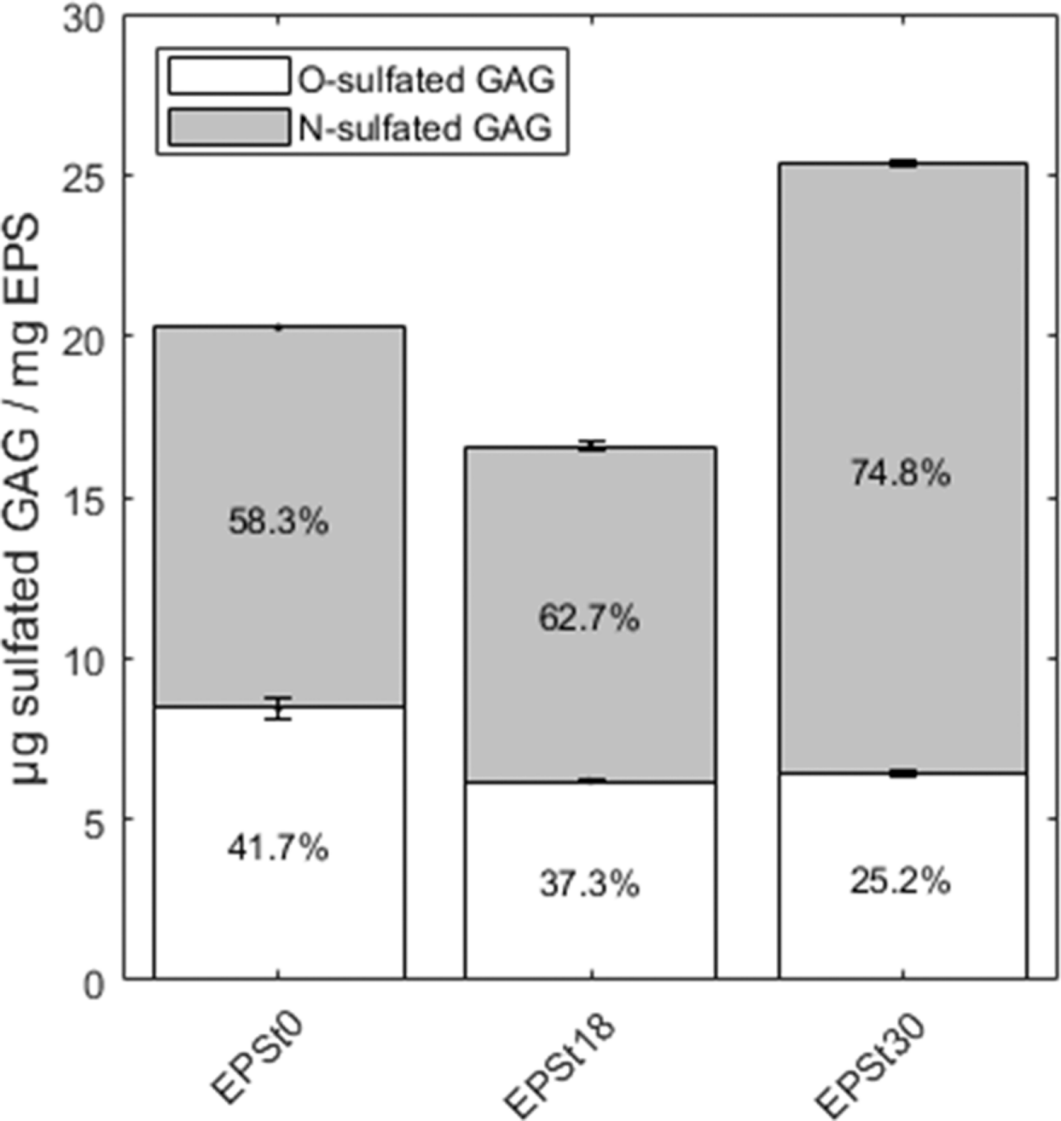
O- and N-sulfated glycosaminoglycan-like polymers (O-sulfated GAGs and N-sulfated GAGs) in the EPS extracted from aerobic granular sludge. EPS_t0_: EPS extracted from inoculum; EPS_t18_: EPS extracted from granules 18 days after the reactor start; EPS_t30_: EPS was extracted from granules 30 days after the reactor start.

The presence of sulfated GAGs was investigated by using the DMMB assay. The following sulfated GAGs, either still attached to the peptide/protein core or as free chains, can be assayed: chondroitin sulfates (4- and 6-sulfated), keratan sulfates (alkali sensitive and resistant forms), dermatan sulfate, and heparan sulfates (including heparins). The total content of sulfated GAGs measured in EPS_t0_, EPS_t18_, and EPS_t30_, was 20.3 ± 0.3, 16.6 ± 0.1, and 25.3 ± 0.2 mg/g, respectively. It seemed that during adaptation to the seawater condition, the amount of these polymers in the EPS was increased. In addition, the percentage of N-sulfated GAGs in the respective EPS increased during adaptation, with the highest percentage in EPS_t30_ (Figure 3). In comparison to the aerobic granular sludge EPS reported by ref (25), the total sulfated GAG content in the EPS of the seawater-adapted granules is much lower, mainly half of the reported amount. Likely, the differences in the operational conditions and microbial communities are the causes.

#### Lectin Microarray

To evaluate protein glycosylation and monitor the dynamic glycan profile of those glycoproteins, a lectin microarray has been used. It is based on the mechanism that lectins selectively bind with glycans by recognizing their specific patterns. It is worth noting that, in this analysis, proteins in the extracted EPS were fluorescently labeled with Cy3. If the labeled proteins are glycosylated and their distinct glycan structures match with the affinity of the lectins, they will bind with the lectins on the microarray and their fluorescent signal will be recorded by the evanescent-field fluorescence scanner. Thus, a strong fluorescent signal indicates the following: the bound proteins are glycoproteins; the glycan part of the bound protein has the same glycan profile pattern that the lectin can recognize, and the amount of this glycoprotein is high.

It was found that for all of the EPS samples, within the 97 lectins used in the lectin microarray, 65 gave a strong fluorescent binding signal. This clearly indicates that there are glycoproteins in all of the EPS samples since only glycoproteins can be detected by the microarray. In addition, from the specificity of the lectins, information on the glycan pattern can be obtained. The result of the lectin microarray showed that there were glycoproteins with N-linked glycosylation (e.g., due to the binding of lectins TxLcl, rXCL, CCA, and rSRL) and O-linked glycosylation (e.g., due to the binding of lectins HEA, MPA, VVA, and SBA). Those glycoproteins contained one or multiple glycans, such as sialic acids (with both α2–3 and α2–6 linkages), lactosamine and/or polylactosamine, mannose (including α1–3 and α1–6 linkages), fucose (including α1–2, α1–3, and α1–4 linkages), *N-*acetyl glucosamine, and galactose (with and without sulfation) (for details of the lectins, refer to the Supporting Information).

**Figure 4:**
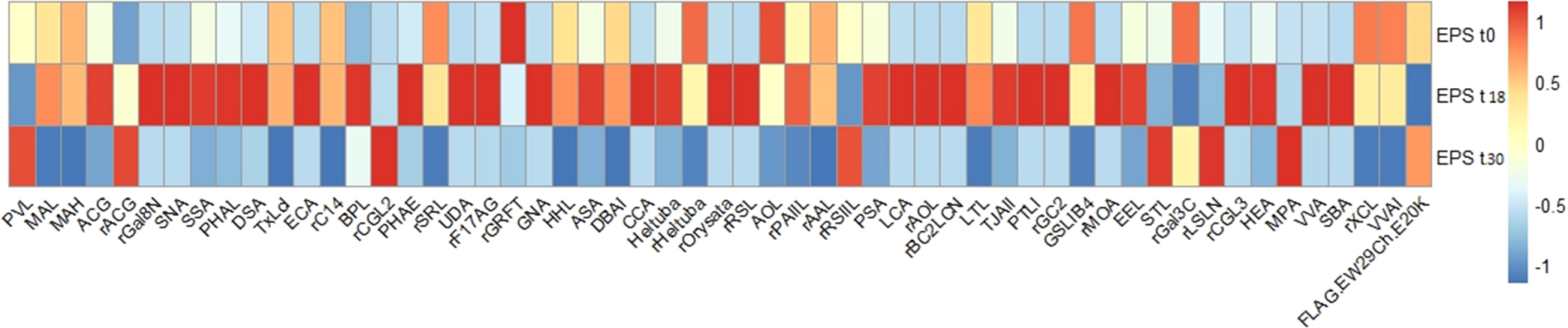
Lectin binding profiles of the extracellular polymeric substances extracted from aerobic granular sludge. EPS_t0_: EPS extracted from inoculum; EPS_t18_: EPS extracted from granules 18 days after the reactor start; EPS_t30_: EPS extracted from granules 30 days after the reactor start. The scale ranges from 1 to −1. Red: high intensity; blue: low intensity.

**Figure 5:**
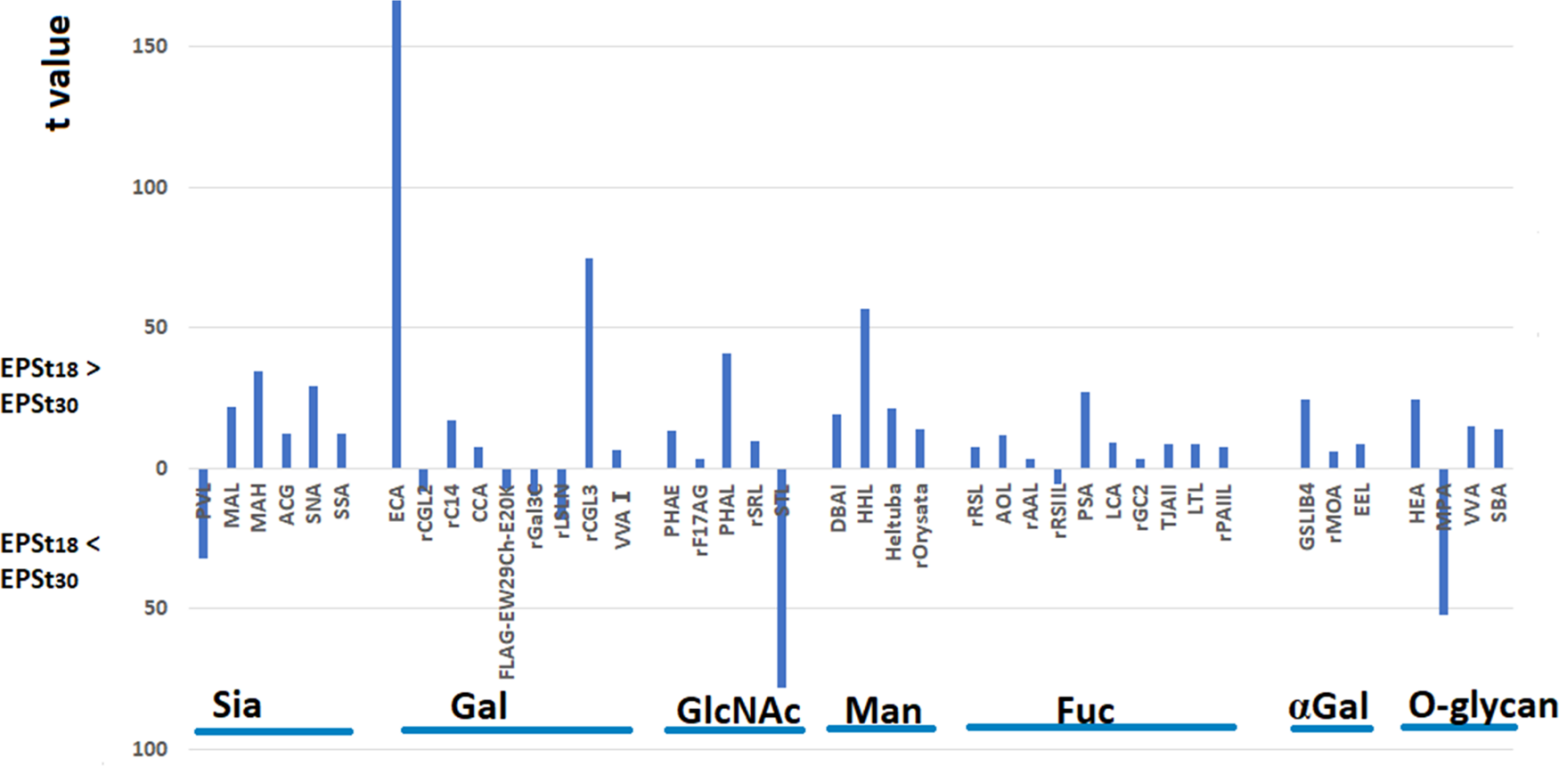
Comparison of the lectin binding profiles of the glycoproteins in the extracellular polymeric substances extracted from aerobic granular sludge. EPS_t18_: EPS extracted from granules 18 days after the reactor start; EPS_t30_: EPS extracted from granules 30 days after the reactor start. Abbreviations: Sia (sialic acid), Gal (galactose), GlcNAc(*N-*acetyl glucosamine), Man (mannose), Fuc (fucose), αGal (terminal α-D-galactose), and O-glycan (O-linked glycoprotein).

Interestingly, 55 lectins were found to be significantly different between the three EPS samples, indicating that the glycan profile of the glycoproteins is altered with the change of the environmental conditions (implied by the color change in Figure 4 from blue to red). If the two EPS extracted from seawater-adapted granules are compared, Figure 5 clearly shows that most of the glycan signals are increased in EPS_t18_, meaning that there are more glycosylated proteins in the EPS_t18_. In addition, as each lectin has its binding specificity, this also shows that the glycan profile of EPS_t18_ has extremely strong diversity, while EPS_t30_ has less glycan diversity. It suggests that, in response to exposure to seawater, the amount of glycoproteins and their glycan diversity first increases; once the granules reach a stable state of adaptation, both the amount of glycoproteins and their glycan diversity tend to decrease. Such a change may also be related to the shift of microbial community; as seen in Figure 2, at t18, the microbial community was more diverse with the presence of PAO, GAO, and other eubacteria; while at t30, PAO was fully dominating over GAO and other eubacteria.

## Discussion

### In Response to the Exposure to Seawater, the Glycan Profile, Especially That of the Glycoproteins in the EPS of Aerobic Granular Sludge, Varied Significantly

During the adaptation to seawater, EPS from aerobic granular sludge exhibited the following variation: there was more EPS, which can be extracted under alkaline conditions (with NaOH present). The yield of the EPS on day 30 was about 2 times that of the inoculum. This is in line with the reported finding that the adaptation of aerobic granular sludge to high saline conditions led to extra EPS production.^27^ Within the EPS, the percentage of glycans detected by GC-MS was increased, as well. The amount of glycans was tripled on day 30. It is known that bacterial glycans can act as osmoprotection and desiccation protection factors against the salt.^28^ Producing a higher amount of glycans in the EPS might be used by the microorganisms as a strategy to protect themselves from harsh environmental stress factors such as the high salt content in seawater. Looking at the glycosyl composition of the glycans, during the adaptation to seawater condition, xylose and *N*-acetyl glucosamine appeared, while arabinose disappeared from the sugar monomers. This indicates that after being exposed to seawater, there is a significant change in glycan composition produced in the EPS. The role of these two sugar monomers against seawater conditions is unknown and needs further investigation. It is also noticed that, in the three EPS samples, the amount of glucose is extremely high in comparison to that of all of the other monomers. The possible explanation could be that there might be glucose-rich glycans, such as β glucan or lipopolysaccharides produced as part of the EPS.^29^ Further investigation is needed to understand the high glucose content.

Within the glycans, besides free polysaccharides, there are glycoconjugates, such as glycoproteins and glycolipids. To further investigate the potential existence of glycoproteins and their glycan profile, lectin microarray analysis was performed. The existence of glycoproteins with diverse glycosylation patterns was observed for all EPS samples, strongly confirming that protein glycosylation is indeed common in aerobic granular sludge. Interestingly, there were more glycoproteins in EPS_t18_ than EPS_t0_ and EPS_t30_, and the glycosylation pattern of EPS_t18_ is significantly diverse. This indicates that, in response to the environmental change, i.e., exposure to the increased salt condition, one of the adaptation strategies of the microorganisms can be altering the glycosylation of proteins in quantity and diversity. Once the steady state of adaptation was reached, the diversity of protein glycosylation and the amount of glycoproteins reduced. In fact, similar phenomena were reported in anaerobic granular sludge: a significant shift in the glycoconjugate pattern in anaerobic granular sludge happened with increasing salinity.^30^ Therefore, it seems that not only the total glycome profile of the EPS but also the glycan profile of glycoproteins are dynamic and sensitive to environmental stimuli such as salinity.

### It Is Important to Investigate the Glycan Profile of Glycoproteins in Aerobic Granular Sludge

The glycome is defined as the entire complement of glycan structures (including glycoproteins/glycolipids and free polysaccharides) produced by cells.^31^ Unlike DNA replication, RNA transcription, or protein translation, glycan biosynthesis is not directed by a pre-existing template molecule.^32^ Instead, the glycome depends on the interplay among the glycan biosynthetic machinery, the available nucleotide sugars (serving as monosaccharide donors), and signals from the intracellular and extracellular environments. Thus, the glycome composition is dynamic and is influenced by both genetic and environmental factors.^33^

In granular sludge, the EPS is produced by the microorganisms and is involved in bacterial cells’ interactions with their environment. As the extracellular environmental condition is one of the factors that influence the glycome, a change in the environmental condition must have its own reflection in the glycan profile. As demonstrated in the current research, the glycan profile, especially the glycoproteins in the EPS, is sensitive to environmental stimuli. Due to the fact that protein glycosylation is an important post-translational modification, small changes in the glycans of glycoproteins can have profound consequences for protein function.^32^ Such sensitivity and dynamic alteration of the glycan profile in the EPS may influence the chemical and physical structures and properties of the EPS and, furthermore, the stability of granular sludge. Further research is needed to find the correlation among the glycan profile dynamics, the property alteration of EPS, and the activities of the microbial community.

### Lectin Microarray Can Be Used as a High-Throughput Approach to Monitor the Diversity and Dynamic Change of the Glycoproteins in the Environmental Sample

Given the profound impact of glycans on the function of glycoproteins, protein glycosylation might play an important role in the EPS of biofilm. However, protein glycosylation in the EPS remains largely uncharacterized, and the existence of glycoconjugates such as glycoproteins (and glycolipids) in the EPS was very recently reported and started getting attention.^33^ On the other hand, the complexity of glycosylation poses an analytical challenge. Current methods for bacterial glycan analysis include MS, HPLC, and HPAEC-PAD. These methods require the release of glycans from a glycoprotein through enzymatic or chemical reactions. This makes an accurate assessment of glycosylation depend on a complete release of all of the glycans that are present in a glycoprotein. In this respect, a significant investment of time and effort is needed, which becomes one of the bottlenecks for a high-throughput study of the diversity and dynamic change of the glycan profile. Recently, using a lectin microarray as a high-throughput approach has attracted great interest. Importantly, the lectin microarray directly measures glycan profiles on an intact protein without the need for enzymatic digestion or clipping glycans from the protein backbone. Such a platform is unique in increasing the possibility of full coverage of all glycan variants of glycoproteins.^34^

In the current work, the application of the lectin microarray indeed confirmed the presence of glycoproteins and effectively monitored its alteration along the adaptation to the seawater condition. Additionally, the result of lectin microarray is in line with the result of other analyses performed: i.e., sugar monomers such as mannose, fucose, galactose, and *N-*acetyl glucosamine were detected by the glycosyl composition analysis through GC-MS. The sialic acids captured by the MS and sulfated glycosaminoglycan-like polymers revealed by the DMMB assay were in line with the presence of sialic acids, lactosamine, and galactose with sulfation (e.g., keratan sulfate) detected by the lectin microarray analysis. This suggests that the lectin microarray is a successful platform for glycan profiling of glycoproteins in microbial aggregates such as granular sludge.

Despite the success, it is worth noting that as lectins are of diverse specificity, some have cross-reactivity with various glycans. It is relatively difficult to characterize a specific glycan using only one lectin. A second limitation is the lack of availability of lectins that recognize sugars unique to bacteria. Designing a bacteria (or biofilm)-specific lectin microarray is an interesting topic for future research.

## Conclusions

Protein glycosylation was identified in the extracellular polymeric substances (EPS) in aerobic granular sludge. In response to environmental stimuli such as exposure to seawater, the glycan profile, especially that of the glycoproteins, varied significantly: xylose and *N*-acetyl glucosamine appeared as sugar monomers in comparison to the inoculation. The amount of glycoproteins and their glycan diversity displayed an increase during adaptation, followed by a decrease once the granules reached a stable state of adaptation. Lectin microarray can be used as a high-throughput approach to monitor the diversity and dynamic change of glycans in the glycoproteins in the EPS of aerobic granular sludge.

## Supporting Information Available

The GC chromatogram of EPS samples and the results of lectin microarray (PDF)
