# Supplementary material for: Alterations of Glycan Composition in Aerobic Granular Sludge during the Adaptation to Seawater Conditions
Source: ACS ES T Water. 2023 Dec 27;4(1):279–86. doi: 10.1021/acsestwater.3c00625 (PMC10788855; doi:10.1021/acsestwater.3c00625)
Supplement: Supplementary file 1 — ew3c00625_si_001.pdf [file ew3c00625_si_001.pdf]

# Alterations of glycan composition in aerobic granular sludge during the adaptation to seawater conditions

Le Min Chen[1], Sunanda Keisham[2], Hiroaki Tateno[2], Jitske van Ede[1], Mario Pronk[1,3], Mark C.M. van Loosdrecht[1], Yuemei Lin[1*]

[1]Department of Biotechnology, Delft University of Technology, Van der Maasweg 9, 2629 HZ Delft, the Netherlands

[2]Cellular and Molecular Biotechnology Research Institute, National Institute of Advanced Industrial Science and Technology (AIST), Central 6, 1-1-1 Higashi, Tsukuba, Ibaraki 305-8566, Japan

[3]Royal HaskoningDHV, Laan 1914 35, Amersfoort 3800 AL, The Netherlands

*Corresponding author

Yuemei Lin

Email address: Yuemei.Lin@tudelft.nl

Supporting information I : the GC-Chromatogram of EPS samples.

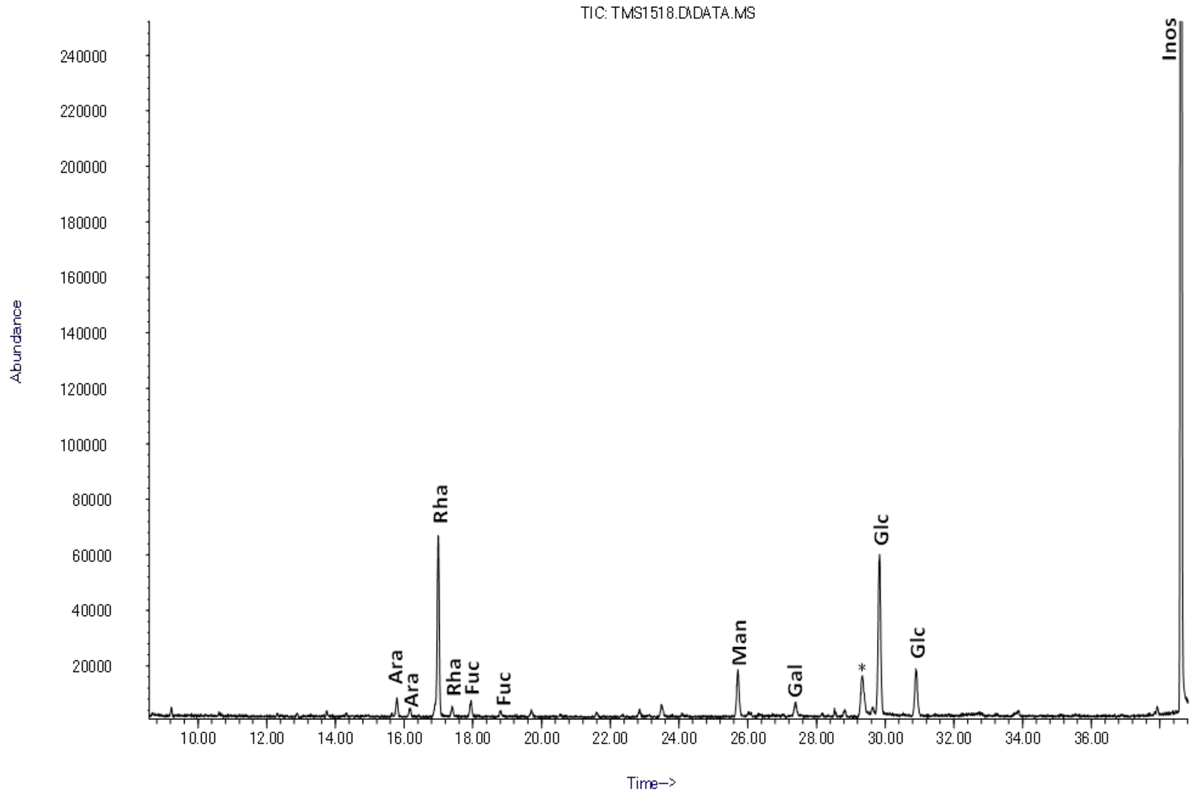


Figure S1: Chromatogram of EPS sample t0 used for composition analysis of TMS methyl glycosides. The asterisk marks an unidentified sugar.

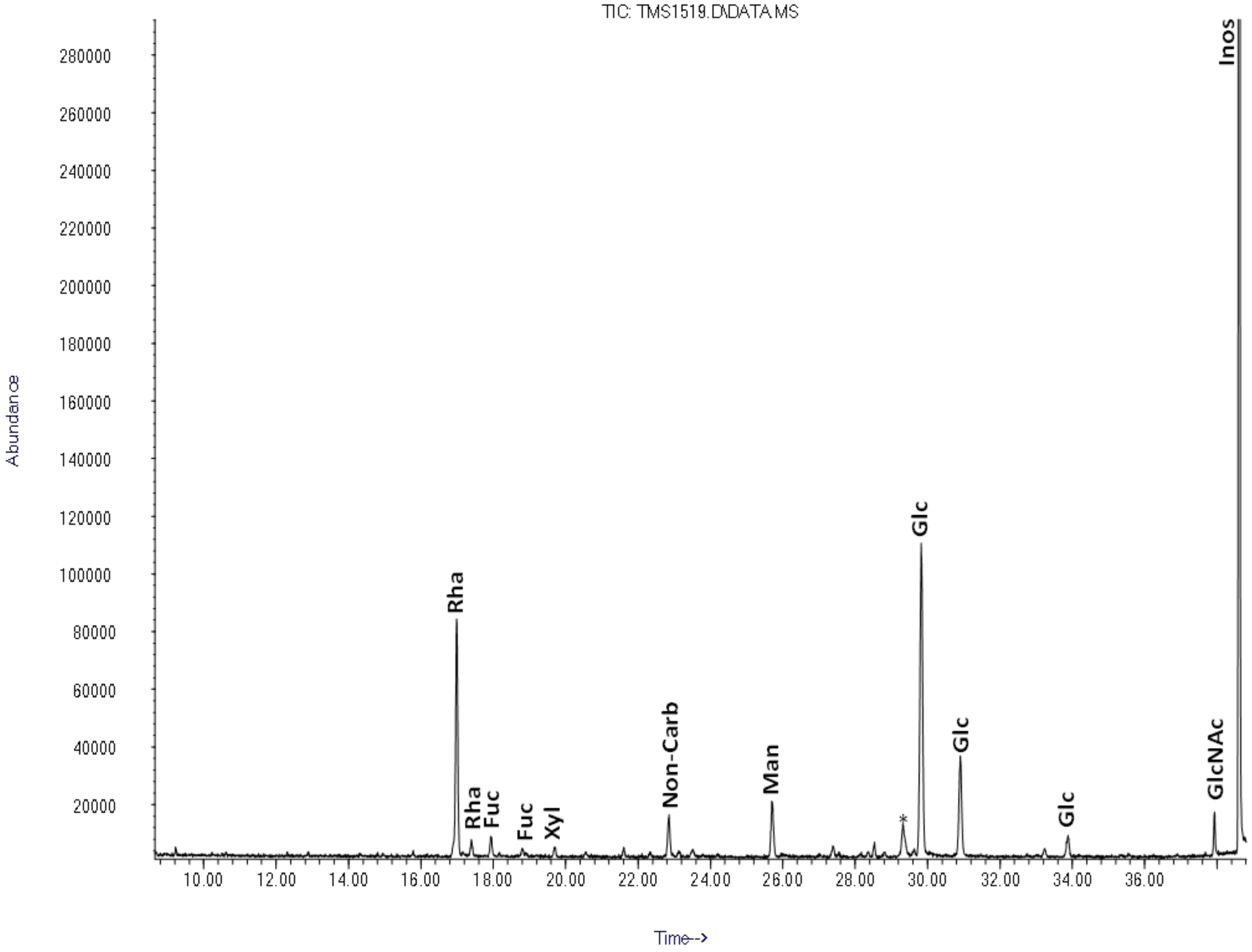


Figure S2: Chromatogram of EPS sample t18 used for composition analysis of TMS methyl glycosides. The asterisk marks an unidentified sugar.

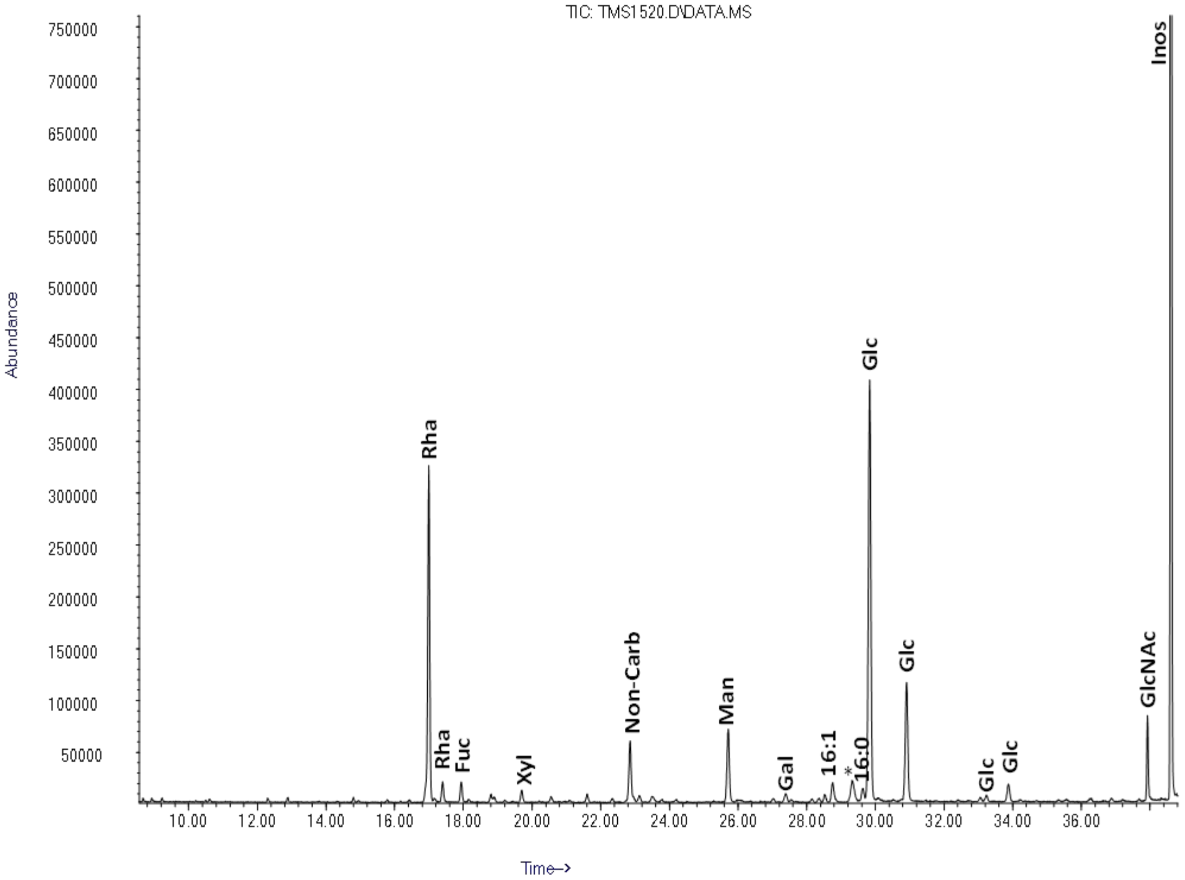


Figure S3: Chromatogram of EPS sample t30 used for composition analysis of TMS methyl glycosides. The asterisk marks an unidentified sugar.

Supporting information II: original data of lectin microarray analysis

Unpaired Student`s t-test was performed

| lectins | Rough glycan specifivity | EPSt1 | EPSt1 | EPSt2 | EPSt2 | p-value | t-value |
|---|---|---|---|---|---|---|---|
| PVL | Sia, GlcNAc | 987,8467 | 977,4472 | 1371,192 | 1349,858 | 0,004876 | -31,8435 |
| MAL | α2-3Sia | 32,68298 | 34,06253 | 6,063929 | 3,89008 | 0,004408 | 22,05803 |
| MAH | α2-3Sia | 53,5288 | 54,04151 | 22,68253 | 24,35789 | 0,01 | 34,54788 |
| ACG | α2-3Sia | 16,18617 | 19,02916 | 0 | 0 | 0,051284 | 12,3867 |
| rACG | α2-3Sia | 962,4884 | 988,3374 | 1099,545 | 1147,125 | 0,054483 | -5,46359 |
| rGal8N | α2-3Sia | 0,588588 | 1,031702 | 0 | 0 | 0,169946 | 3,6566 |
| SNA | α2-6Sia | 15,95727 | 17,09677 | 0 | 0 | 0,021938 | 29,00758 |
| SSA | α2-6Sia | 84,83841 | 79,34277 | 24,34153 | 16,09895 | 0,010424 | 12,49064 |
| | | | | | | | |
| ECA | βGal | 6,785111 | 6,730625 | 0 | 0 | 0,002566 | 248,0588 |
| BPL | Galβ1-3GlcNAc(GalNAc), a/βGalNAc | 65,08802 | 56,82547 | 47,93936 | 50,5112 | 0,191329 | 2,711363 |
| rCGL2 | GalNAcα1-3Gal (A), PolyLacNAc | 779,9771 | 735,1283 | 1017,51 | 1070,73 | 0,015677 | -8,23505 |
| | | | | | | | |
| | | | | | | | |
| rGRFT | Man | 393,4383 | 387,9362 | 345,6725 | 325,6895 | 0,095206 | 5,307769 |
| GNA | Manα1-3Man, Manα1-6Man | 4,185514 | 4,781855 | 0 | 0 | 0,042274 | 15,03732 |
| HHL | Manα1-3Man, Manα1-6Man | 230,4158 | 224,8618 | 65,33025 | 66,64007 | 0,007365 | 56,65737 |
| ASA | Galβ1-4GlcNAcβ1-2Man | 116,4587 | 115,9927 | 48,33981 | 51,25929 | 0,011795 | 44,93681 |
| DBAI | High-man | 67,04998 | 60,46099 | 0 | 0 | 0,032867 | 19,35214 |
| Heltuba | Manα1-3Man | 24,4264 | 22,22253 | 0,028603 | 0 | 0,030046 | 21,15203 |
| rHeltuba | Manα1-3Man | 151,6922 | 132,9749 | 113,9847 | 115,9244 | 0,206478 | 2,909942 |
| rOrysata | Manα1-3Man, Highman, biantenna | 7,275601 | 6,288467 | 0,028603 | 0 | 0,046177 | 13,70613 |
| | | | | | | | |
| rRSL | αMan, α1-2Fuc (H), α1-3Fuc (Lex), α1-4Fuc (Lea) | 1,684015 | 1,293721 | 0 | 0 | 0,082969 | 7,629463 |
| AOL | α1-2Fuc (H), α1-3Fuc (Lex), α1-4Fuc (Lea) | 25,80217 | 30,55183 | 0 | 0 | 0,053529 | 11,86485 |
| rPAIIL | αMan, α1-2Fuc (H), α1-3Fuc (Lex), α1-4Fuc (Lea) | 62,49437 | 57,77588 | 0,276376 | 13,93351 | 0,057213 | 7,340187 |
| rAAL | α1-2Fuc (H), α1-3Fuc (Lex), α1-4Fuc (Lea) | 79,30372 | 66,80336 | 22,57068 | 42,64784 | 0,096785 | 3,420147 |
| rRSIIL | α1-2Fuc (H), α1-3Fuc (Lex), α1-4Fuc (Lea) | 220,95 | 208,1277 | 307,2376 | 343,1598 | 0,074385 | -5,80251 |
| PSA | α1-6Fuc up to biantenna | 23,29784 | 21,63055 | 0 | 0 | 0,023614 | 26,94699 |
| LCA | α1-6Fuc up to biantenna | 41,01786 | 32,92375 | 0 | 0 | 0,069412 | 9,135243 |

| | | | | | | | |
|---|---|---|---|---|---|---|---|
| rAOL | α1-2Fuc (H), α1-3Fuc (Lex), α1-4Fuc (Lea) | 5,881376 | 3,7526 | 0 | 0 | 0,138446 | 4,525593 |
| rBC2LCN | Fuc α1-2Galβ1-3GlcNAc (GalNAc) | 0,569165 | 0,354019 | 0 | 0 | 0,145762 | 4,290955 |
| TJAII | α1-2Fuc | 67,00975 | 84,25649 | 0 | 0 | 0,072273 | 8,770715 |
| rGC2 | α1-2Fuc (H), αGalNAc (A), αGal (B) | 0,265611 | 0,495626 | 0 | 0 | 0,186808 | 3,309498 |
| | | | | | | | |
| GSLIB4 | αGal (B) | 77,10295 | 71,15779 | 0,092125 | 0,753163 | 0,02397 | 24,64401 |
| rMOA | αGal (B) | 1,138331 | 0,814243 | 0 | 0 | 0,104711 | 6,024836 |
| EEL | αGal (B) | 51,22489 | 64,67924 | 0 | 0 | 0,073571 | 8,61462 |
| | | | | | | | |
| PHAE | bisecting GlcNAc | 22,51349 | 19,34031 | 0 | 0 | 0,048174 | 13,18987 |
| UDA | (GlcNAc)n | 0,523189 | 0,47491 | 0 | 0 | 0,03077 | 20,67357 |
| rF17AG | GlcNAc | 2,501499 | 1,375602 | 0 | 0 | 0,179923 | 3,443568 |
| PHAL | GlcNAcβ1-6Man (Tetraantenna) | 29,95259 | 31,45871 | 0 | 0 | 0,01561 | 40,77436 |
| DSA | GlcNAcβ1-6Man (Tetraantenna) | 21,74505 | 23,81102 | 0 | 0 | 0,028851 | 22,05076 |
| | | | | | | | |
| TxLcI | Galactosylated N-glycans up to triantenna | 74,78337 | 75,70725 | 15,30284 | 15,73986 | 0,000821 | 116,8731 |
| rXCL | Core1,3, αGalacto N-glycan | 5,274267 | 4,637647 | 0 | 0 | 0,040833 | 15,5696 |
| CCA | Galactosylated N-glycans up to triantenna | 7,945937 | 6,124705 | 0 | 0 | 0,081945 | 7,725891 |
| rSRL | Core1,3, agalacto N-glycan | 38,99395 | 40,77678 | 15,50306 | 10,11421 | 0,043108 | 9,540593 |
| | | | | | | | |
| rC14 | Branched LacNAc | 44,34029 | 39,3848 | 0,114414 | 0 | 0,037592 | 16,86783 |
| STL | Polylactosamine, (GlcNAc)n | 89,73842 | 93,24857 | 280,7055 | 284,1306 | 0,000166 | -77,8596 |
| rGal3C | LacNAc, polylactosamine | 877,6531 | 817,5711 | 1149,078 | 1141,795 | 0,060622 | -9,84187 |
| rLSLN | LacNAc, polylactosamine | 193,4024 | 209,1189 | 541,6041 | 510,0794 | 0,010285 | -18,4289 |
| rCGL3 | LacDiNAc | 15,78486 | 16,21406 | 0 | 0 | 0,008539 | 74,55342 |
| | | | | | | | |
| LTL | Lex, Ley | 82,52899 | 104,1523 | 0 | 0 | 0,073413 | 8,63332 |
| PTLI | αGalNAc (A, Tn) | 2,08694 | 1,840898 | 0 | 0 | 0,039826 | 15,9641 |
| HEA | Galβ1-3GalNAc (T) | 1681,694 | 1725,24 | 880,0722 | 826,5961 | 0,002009 | 24,65465 |
| MPA | Galβ1-3GalNAc (T), GalNAcα (Tn) | 395,2664 | 397,2092 | 765,3763 | 779,7117 | 0,010663 | -52,0247 |
| VVA | a,βGalNAc (A, Tn, LacDiNAc) | 10,01731 | 8,779668 | 0 | 0 | 0,041856 | 15,1877 |
| SBA | a,βGalNAc (A, Tn, LacDiNAc) | 10,20703 | 8,850471 | 0 | 0 | 0,04524 | 14,04838 |
| VVA I | GalNAcβ1-3(4)Gal | 69,17258 | 67,40519 | 23,95256 | 35,77523 | 0,091175 | 6,428784 |
| FLAG-EW2[illegible] | 6-sulfo-Gal | 643,4985 | 586,5738 | 829,0348 | 821,4181 | 0,081048 | -7,31963 |
